# Diffeomorphic Registration of Images with Variable Contrast Enhancement

**DOI:** 10.1155/2011/891585

**Published:** 2010-12-08

**Authors:** Guillaume Janssens, Laurent Jacques, Jonathan Orban de Xivry, Xavier Geets, Benoit Macq

**Affiliations:** ^1^Institute of Information and Communication Technologies, Electronics and Applied Mathematics (ICTEAM), Universitè Catholique de Louvain (UCL), 1348 Louvain-la-Neuve, Belgium; ^2^Department of Radiation Oncology and Laboratory of Radiobiology and Radiation Protection (RBNT), Universitè Catholique de Louvain (UCL), 1200 Brussels, Belgium

## Abstract

Nonrigid image registration is widely used to estimate tissue deformations in highly deformable anatomies. Among the existing methods, nonparametric registration algorithms such as optical flow, or Demons, usually have the advantage of being fast and easy to use. Recently, a diffeomorphic version of the Demons algorithm was proposed. This provides the advantage of producing invertible displacement fields, which is a necessary condition for these to be physical. However, such methods are based on the matching of intensities and are not suitable for registering images with different contrast enhancement. In such cases, a registration method based on the local phase like the Morphons has to be used. In this paper, a diffeomorphic version of the Morphons registration method is proposed and compared to conventional Morphons, Demons, and diffeomorphic Demons. The method is validated in the context of radiotherapy for lung cancer patients on several 4D respiratory-correlated CT scans of the thorax with and without variable contrast enhancement.

## 1. Introduction

In the context of image-based medical diagnostics and treatment, highly deformable anatomies are a problem for multiple-time imaging analysis along the course of treatment. Indeed, a precise tracking of organs is made difficult because of shape and position variations. Nonrigid registration may be used to compute a displacement vector for each voxel of an image [1], enabling the estimation of the spatial variations of the anatomy. The displacement vectors are computed as pointing to the best corresponding location of the voxels in another image according to a metric which is a measure of the image matching and under some constraints on global properties of the resulting deformation, such as invertibility and smoothness.

Several registration methods have been used in the past years to estimate deformations in highly deformable anatomies [2–5]. Many efforts have been made to improve the quality of displacement estimates and also to reduce the amount of required preprocessing or modeling and improve registration speed [6–8]. Besides, the choice of a registration method for medical application depends on the characteristics (e.g., modality) of the images to be registered [1]. The existing methods [9] can be divided into parametric, or model-based, methods (B-splines [10], thin-plate splines [11], radial basis functions [12], linear elastic FEM [13], etc.) and nonparametric methods (viscous fluid [14], optical flow [15], etc.). In this second category, the algorithm called *Demons* [16, 17] is fast, efficient, and easy to use, as it requires no particular preprocessing nor patient-specific modeling. This method aims at calculating a regular displacement field which produces a good matching of the intensities in both images by minimizing a metric, such as the sum of squared differences (SSDs) [18] or the mutual information (MI) [8] between images along with a measure of the field regularity.

In a growing number of applications, the displacement fields resulting from registration are used to deform images from other modalities or other spatial distribution maps (e.g., the dose map associated to CT scans in radiotherapy [19, 20]). Therefore, the matching of structures in images based on their intensities is not a sufficient constraint for producing realistic anatomical deformation estimations [21]. This is the reason why a priori information on the physical characteristics of anatomical deformations has to be included in the registration process. Diffeomorphism is a necessary condition for displacement fields to be physical [22]. Indeed, organs can be compressed and deformed, but cannot undergo noninvertible spatial transformations, for example, showing mirror effects. A method has been proposed in [23] to limit the displacement fields computed by the Demons to a set of diffeomorphic transformations, using diffeomorphic flows and Lie algebra.

In several medical protocols, contrast agents are used in order to facilitate interpretation. This makes the registration problem incompatible with the hypothesis of intensity conservation. Furthermore, an histogram equalization is often not able to correct for contrast agent variability, as different regions will be enhanced in different ways inside the image. Therefore, simple metrics, such as SSD or cross-correlation, are not suitable for matching those images, and methods that are suitable for registering variable contrast images have to be investigated [24, 25].

A method similar to Demons but using a phase-based approach was first proposed in [26] and was called *Morphons*. The principle of the method is to match transitions (between dark and bright zones) rather than intensities, by looking locally at the spatial oscillations in intensities. This method uses Gaussian smoothing as regularization of the displacement field and additive accumulation during the iterative process. This is nevertheless not sufficient to ensure the invertibility of the deformation [22, 27].

In this paper, a Morphons registration using a diffeomorphic accumulation step is proposed and its accuracy is assessed in the case of thorax image registration, also in presence of different contrast enhancements, and compared to the Demons. The paper is organized as follows. In Section 2, the main mathematical concepts and definitions are presented. Then, in Section 3 a generic nonparametric registration process is presented, and its particularization to Morphons and to diffeomorphisms is proposed in Section 4. In Section 5, different registrations are applied on images of the thorax, without contrast enhancement in the first experiment and with contrast enhancement in the second. The results of these experiments are eventually discussed in Section 6.

## 2. Mathematical Framework

For the sake of clarity, let us introduce some key mathematical concepts used throughout this paper.

### 2.1. Images and Deformation Fields

In this paper, we always denote 3D images by lower case letters. For instance, in the process of estimating a displacement field, the fixed and the moving images are written *f* and *m*, respectively. We consider them as real valued functions on the volume ℝ^3^ of points *x* = (*x*_1_, *x*_2_, *x*_3_), that is, *f*, *m* ∈ *ℱ* = {*g* : ℝ^3^ → ℝ : *x* ↦ *g*(*x*)}. Most of the time, these functions, and also the continuous operations performed on them, such as convolutions or integrals, must be understood as approximated on the discrete *voxel* grid *𝒢* = {(*x*_1_, *x*_2_, *x*_3_) ∈ *ℤ*^3^}, omitting the treatment of volume boundaries. In this study, image convolutions were performed using zero-padding outside the boundaries.

A displacement field on ℝ^3^ is a vectorial field *D* ∈ *𝒱* = {*V* : ℝ^3^ → ℝ^3^, *x* ↦ *V*(*x*)}. It is associated to the “deformation” operation Δ ≜ Id + *D*, that is, Δ(*x*) ≜ *x* + *D*(*x*), with Id the identity deformation: Id(*x*) ≜ *x*. The operation Δ, and by extension its vector field *D*, is said to be *diffeomorphic* if it is invertible, differentiable, and its inverse is differentiable. For the transformation Δ to be invertible, its Jacobian must not vanish in any point *x*, that is, if det (*𝒥*)(*x*) ≠ 0  for  all  *x*, with *𝒥*_*ij*_ = ∂Δ_*i*_/∂*x*_*j*_. Moreover, it has to be positive (det (*𝒥*)(*x*) > 0). Indeed, a transformation Δ with negative Jacobians does not correspond to physical deformations (as the *mirror* operation).

Mathematically, given the images *f* and *m*, we will see that our global objective of our study is to estimate *D* such that the *warping* of *m* by *D* is “close” to *f*, that is, *f*≃*m*∘Δ with ∘ the common function composition. We will use sometimes the notation

(1) $$\begin{array}{c} m⋄D\;=\;m\circ \Delta , \end{array}$$

to insist on the *warping* action of *D* on *m*. By extension, this warping symbol can also be used on vector fields themselves, for example, for two displacement fields *D*_1_ and *D*_2_, *D*_1_⋄*D*_2_ = *D*_1_∘Δ_2_.

In practice, the warping is applied on discrete images. The transformation might therefore need to be truncated (on the volume boundaries) to the closest point inside the volume in order to avoid extrapolation of the images to be warped.

### 2.2. Compositive Accumulation

**Figure 1:**
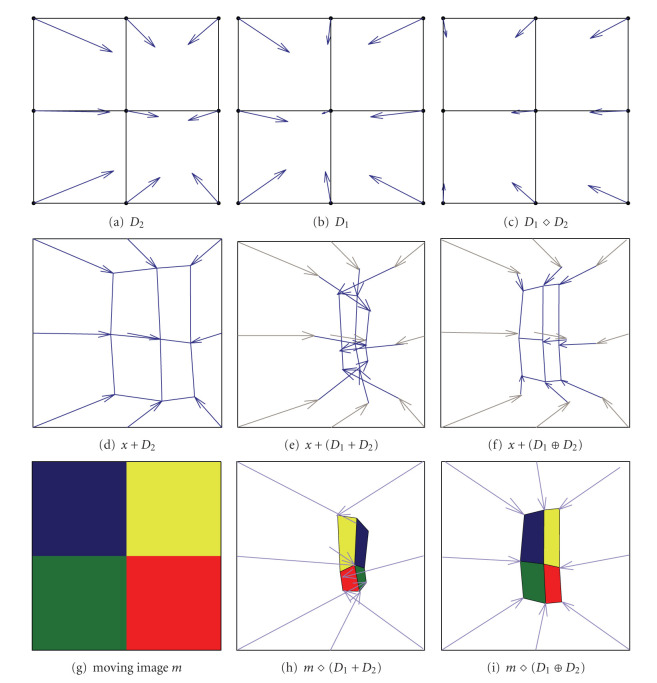
Comparison between additive and compositive field accumulations. Warping is implemented using linear interpolation. In (a) and (b), two different displacement fields are defined on the plane (for visual clarity). In (c), the field *D*_1_ warped by *D*_2_, that is, *D*_1_⋄*D*_2_ = *D*_1_∘Δ_2_. In (d), the field *D*_2_ is applied on the pixel grid. In (e), the grid is warped by the field resulting from an addition-based accumulation of *D*_1_ and *D*_2_. In (f), the grid is warped by the displacement field *D*_1_ ⊕ *D*_2_ arising by the composition of Δ_1_ and Δ_2_, which is the sum of the dark blue and gray arrows (given by Δ_1_∘Δ_2_ − Id). This composition is really the accumulation that matters since it corresponds to the way displacement fields are iteratively applied to an image (see Section 3). Since *D*_1_ ⊕ *D*_2_ = *D*_2_ + *D*_1_⋄*D*_2_, the summed vectors in (f) correspond to the vectors in (a) and (c). In (g), a moving object *m*, divided in 4 colors (regions between pixel centers). In (h), the result of the warping of *m* by the sum of the fields. Clearly, the surfaces are inverted (mirror effect, visible because of the inversion of colors), leading to nonphysical deformations (negative Jacobians). In (i), the result of the warping of *m* by the composition of the fields. One can notice that in spite of the deformation of the shape of the object, the location of the colors is conserved.

In this paper, we promote a particular way to combine, or *accumulate*, properly two displacement fields *D*_1_ and *D*_2_. Adding them to form *D*_1_ + *D*_2_ (as performed by many nonparametric registration methods; see Section 3) is of course computationally efficient, but it breaks the consistency with the composition of the corresponding spatial transformations, as illustrated in Figure 1.

Indeed, one can clearly see in Figure 1(h) that the warping of the image in Figure 1(g) by the sum of two diffeomorphic fields, *D*_1_ and *D*_2_ does not correspond to the successive warping of this image by *D*_1_ and then by *D*_2_, which is represented in Figure 1(i).

However, the *compositive* operation, denoted by ⊕, solves this issue. It is simply defined as

(2) $$\begin{array}{c} D_{1}⊕D_{2}≜\Delta_{1}\circ \Delta_{2}-\text{Id}. \end{array}$$

By construction, the deformation operation linked to the deformation field *D*_1_ ⊕ *D*_2_ is therefore Δ_1_∘Δ_2_. If both displacement fields are diffeomorphic, their composition is also diffeomorphic [28].

The operation ⊕ has some interesting and useful properties. First, the neutral accumulation is of course obtained with the null displacement field, that is, *D* ⊕ 0 = 0 ⊕ *D* = *D*. Second, it is easy to prove the *associative* relations (*D*_1_ ⊕ *D*_2_) ⊕ *D*_3_ = *D*_1_ ⊕ (*D*_2_ ⊕ *D*_3_) = *D*_1_ ⊕ *D*_2_ ⊕ *D*_3_ for three displacement fields *D*_1_, *D*_2_, and *D*_3_. And finally, ⊕ and ⋄ are linked through the simple relation

(3) $$D_{1}⊕D_{2}=D_{2}+D_{1}⋄D_{2},$$

meaning that the displacement field *D*_1_ ⊕ *D*_2_ is equivalent to summing the field *D*_2_ with the field *D*_1_ warped by *D*_2_. This is illustrated in Figure 1: the vectors in Figure 1(i), corresponding to the successive warping by *D*_1_ and then *D*_2_, are the sum of the vectors in Figures 1(a) and 1(c), as shown in Figure 1(f).

### 2.3. Diffeomorphic Flow and Exponentiation

An important notion used in Section 4.2 is the concept of (continuous) *diffeomorphic flow* [27, 29, 30]. Given a point *x* ∈ ℝ^3^ and a smooth vector field *D* ∈ *𝒱*, the flow *φ*_*D*_(*x*, *t*) is the dynamic solution *u*(*t*) ∈ ℝ^3^ of the following (autonomous) ordinary differential equation:

(4) $$\begin{array}{c} \frac{\text{d}}{\text{d}t}u\left(t\right)=D\left(u\right),\\ u\left(0\right)=x. \end{array}$$

**Figure 2:**
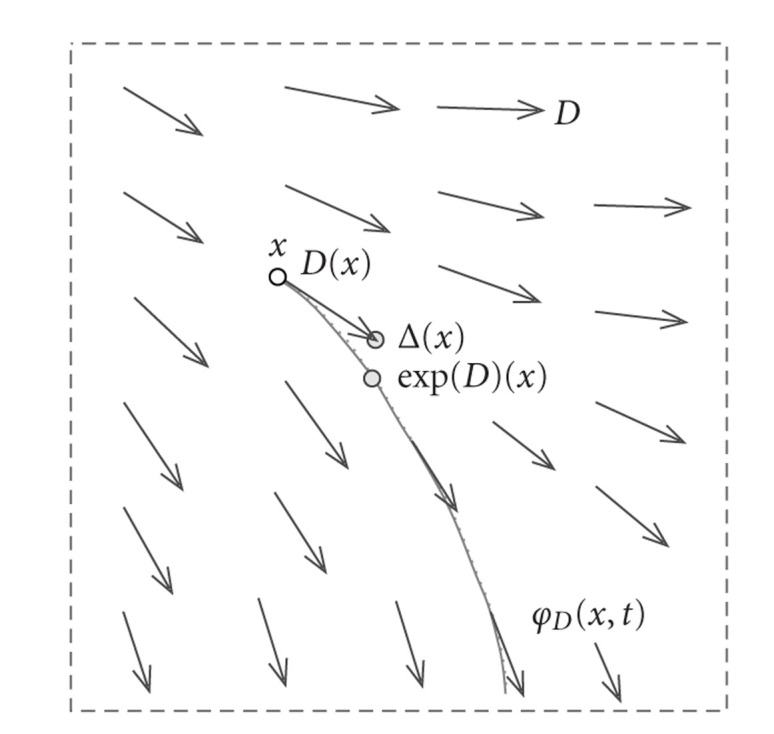
The diffeomorphic flow exp (*D*) associated to the vector field *D* is the solution at time *t* = 1 on the trajectory tangent to *D* at each point (here represented in 2D). We see that the motion of *x* induced by exp (*D*)(*x*) is more compatible with *V* than this produced by Δ(*x*) = *x* + *D*(*x*).

At a given “time” *t* > 0, the position *φ*_*D*_(*x*, *t*) is simply a point on the trajectory following *D* tangentially from the initialization on *x* (see Figure 2). Following [27], the exponential of a vector field *D*, that is, exp (*D*) ∈ *𝒱*, is the nonlinear deformation operation obtained by the flow of *D* at time *t* = 1, that is, exp (*D*)(*x*) = *φ*_*D*_(*x*, 1). Interestingly, this exponential map acts as the common scalar-valued exponential, that is, exp (*αD*)∘exp (*βD*) = exp ((*α* + *β*)*D*) for *α*, *β* ∈ ℝ, and it is invertible by simply considering the inverted vector field, that is, exp (−*D*)∘exp (*D*) = Id. In addition, for differentiable *D*, exp (*D*) is also a diffeomorphism on ℝ^3^. In other words, exp (*D*) modifies the 3D coordinates with no intersection between the motions of points. Indeed, such a possibility would induce a point *x* with two different motion vectors, a situation that is forbidden by (4) since *D*(*x*) is uniquely defined.

### 2.4. Scaling and Squaring

A numerical scheme exists to compute approximately but efficiently exp (*D*)(*x*) when *x* belongs to a regular grid of voxels *𝒢*. Indeed, when the field *D* is close enough to zero (i.e., Δ ≈ Id), the exponential of the field can be approximated using the first-order Taylor expansion exp (*D*) ≈ Id + *D* = Δ, that is, by the transformation itself. On the other hand, the solution of the flow equation (4) in *t* = 1 can be approximated by “discretizing” *t* between 0 and 1. Indeed, as exp (*D*) = exp (2^−*k*^*D*)^2^*k*^^ (where the exponent 2^*k*^ expresses the number of times the deformation operation is combined with itself), one can use the *scaling and squaring* strategy for computing the exponential [31]. If one chooses *k* such that the field 2^−*k*^*D* is close enough to zero, the first-order approximation can be used to estimate exp (2^−*k*^*D*) (based on the Padé approximant near the origin). Then, the solution of the flow equation is computed by performing *k* recursive compositions of the field by itself, given that such compositions are computationally affordable. Notice that taking *k* = 0 is equivalent to the simple first-order approximation. The scaling and squaring steps for field exponentiation [22] are depicted hereafter.

- *Scaling.* Divide *D* by a factor 2^*k*^ such that 2^−*k*^*D* is small enough, for example, when ||2^−*k*^*D*||_*∞*_ = max_*x*_||2^−*k*^*D*(*x*)|| < 0.5 voxels.
- *Exponentiation.* Compute first-order explicit integration of the flow: Δ^(0)^(*x*) = *φ*_*D*_(*x*, 2^−*k*^) ≈ Id(*x*) + 2^−*k*^*D*(*x*).
- *Squaring.* Perform *k* recursive squarings (using field composition) of the flow at time 2^−*k*^ in order to obtain the flow at time 1, which is the field exponential. In other words, starting with Δ* = Δ^(0)^, do *k* times the computation Δ* ← Δ*∘Δ*, in order to get Δ*≃exp (*D*).

We see that using this method, only *k* compositions (and therefore *k* interpolations) are needed for estimating the exponential. Compared to standard estimation of the flow over a regular discretization of the time interval [0,1] in 2^*k*^ steps, the scaling and squaring method limits the numerical errors due to composition of vector fields, but it does not decrease the amplification of the error due to the field estimation at time *t* = 2^−*k*^.

## 3. Generic Registration Pipeline

Nonrigid registration methods can be divided into parametric and nonparametric methods. Parametric (or model-based) methods aim at calculating the parameters of a deformation model in a high-dimensional space in order to optimize a global objective function that takes into account image similarity and transformation regularity [10]. In this case, the a priori information is included in the modelization and regularity criteria of the nonrigid transformation. For example, the harmonic energy of transformation can be explicitely included in the objective function [32].

On the other hand, nonparametric methods make it possible to decouple similarity optimization from regularization by directly acting on the displacement field. The a priori information has then to be included in the optimization process by using proper regularization techniques. Decoupled optimization makes the registration computationally efficient [8], mainly because the computation of each displacement vector is independent from others, but it prevents us from easily including more complex regularization constraints in the process, for example, such as in volume preserving registrations [33, 34].

### 3.1. Multiscale Nonparametric Registration

Most nonparametric registrations are based on an iterative process which is composed of 3 steps: (i) field computation, (ii) field accumulation, and (iii) field regularization. The idea is to progressively build a proper displacement field by iteratively improving the matching between the fixed image and the moving image warped by this displacement field, according to a certain metric. Note that, depending on the nature of the displacement one tries to model, the regularization is applied either on the increment field or on the accumulated field. Regularizing the field increment corresponds to a viscous fluid modeling, while regularizing the global transformation corresponds to an elastic solid modeling [14]. Only the second is considered in this study.

In this paper, our general nonparametric registration framework (e.g., valid for Demons and Morphons) adopts a *multiscale* approach; that is, the displacement field estimation is stabilized by decomposing the fixed and the moving images in several *scales*, for example, using a simple smoothing and downsampling procedure [35].

**Figure 3:**
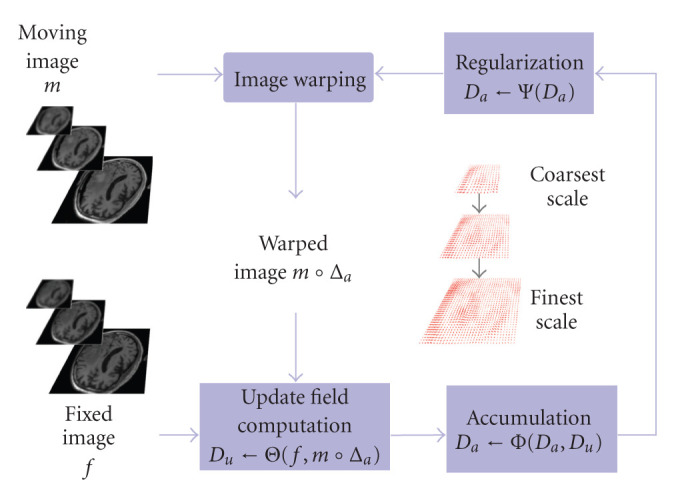
The nonparametric registration pipeline is composed of 3 main operations (Θ, Φ, and Ψ) and the warping of the moving image. Those operations are performed from coarse to fine scales. At each scale, the process is applied iteratively, until it reaches a stopping criterion.

The three steps mentioned above are then applied a certain number of times (until the algorithm reaches a certain stopping criterion) to each scale separately from coarse to fine scales (Figure 3). The general explanation of these three basic blocks is given hereafter. The way they are iteratively applied at each scale is described in Section 3.2.

#### 3.1.1. Field Computation

At each iteration of the registration process, an update displacement field (*D*_*u*_) is first computed as a function (Θ) of the fixed image (*f*) and the moving image (*m*) warped by the displacement field resulting from previous iterations (*D*_*a*_):

(5) $$\begin{array}{c} D_{u}⟵\Theta \left(f,m\circ \Delta_{a}\right), \end{array}$$

where Δ_*a*_ and Δ_*u*_ denote the deformation operations linked to *D*_*a*_ and *D*_*u*_, respectively.

 Depending on the nature of the images to be registered, this local displacement estimation can be based on different local image metrics, such as SSD [17], mutual information computed on blocks of voxels [8, 36], and local phase [26].

#### 3.1.2. Field Accumulation

After the field computation, the total displacement *D*_*a*_ must be increased by the update field

(6) $$\begin{array}{c} D_{a}⟵\Phi \left(D_{a},D_{u}\right). \end{array}$$

This accumulation operation Φ is sometimes implemented as a simple addition of accumulated and update fields (as in [18, 37, 38]). However, as explained in Section 2.2, this accumulation is perhaps computationally efficient but is not consistent with the composition of the corresponding spatial transformations. The solution is, therefore, to replace it by the compositive accumulation ⊕ introduced earlier. The accumulation *D*_*a*_ ⊕ *D*_*u*_ of the displacement fields *D*_*a*_ with *D*_*u*_ is then compatible with the way *D*_*u*_ is estimated. Indeed, since *D*_*u*_ is computed from *m*∘Δ_*a*_, the accumulation of *D*_*a*_ and *D*_*u*_ must modify *D*_*a*_ by *D*_*u*_, a process intrinsically integrated by the operation ⊕. Moreover, the associativity of ⊕ validates the compositive accumulation of displacement fields over several iterations, as illustrated in Figure 3.

#### 3.1.3. Field Regularization

Eventually, the field is regularized in order to get a smoother transformation and reduce the impact of image noise on the registration output:

(7) $$\begin{array}{c} D_{a}⟵\Psi \left(D_{a}\right). \end{array}$$

This operation Ψ is achieved by applying a low-pass filter on each component of the displacement field. We assume it to be a Gaussian smoothing with a size *σ*_Ψ_^2^ of a few voxels, which tends to reduce the harmonic energy of the transformation [32].

It is always possible to produce invertible fields by performing a very strong Gaussian smoothing. This, however, may reduce significantly the accuracy of the estimated displacement by limiting the solution to excessively smooth displacement fields. On the other hand, by preventing the displacement field from being noninvertible, the diffeomorphic accumulation acts in some way as a regularization, allowing the estimation of invertible fields while performing only moderate smoothing.

### 3.2. Registration Algorithm

Let us explain now the whole multiscale nonparametric registration algorithm relying on the three specific procedures {Θ, Φ, Ψ} defined in Section 3.1.

The algorithm takes as inputs the fixed and the moving images *f* and *m*, some parameters described below, and outputs the estimated transformation Δ_*a*_ = Id + *D*_*a*_ such that *f*≃*m*∘Δ_*a*_. The whole procedure described in Table 1 and depicted in Figure 3 involves computations on different scales *j* ∈ [0, *J*], from coarse (*j* = *J*) to fine (*j* = 0). Each scale is associated to a subsampled grid of voxels *𝒢*_*j*_ = *κ*^*j*^*𝒢*, where *κ* is the subsampling factor (e.g., $\kappa =\sqrt{2}$ in this study) between scale *j* and scale *j* + 1. The functions *f* and *g*, defined on the initial grid *𝒢* = *𝒢*_0_ = {(*x*_1_, *x*_2_, *x*_3_) ∈ *ℤ*^3^}, are downsampled (after antialiasing smoothing) at any scale *j* by the operation Down*_j_*(). An upsampling operator Up(), implemented as a simple linear interpolation, is used to transfer any displacement field defined on a grid *𝒢*_*j*+1_ to the finer grid *𝒢*_*j*_ using *κ* as upsampling factor. For each scale *j* ∈ [0, *J*], the accumulated displacement field is iteratively updated until one reaches a particular stopping criterion *𝒮* (e.g., based on the convergence of *D*_*a*_ or on the SSD, as precised in Section 5).

## 4. Diffeomorphic Morphons

Our paper adapts the global registration method explained in the previous section to Morphons [26, 39] by taking care of the invertibility of the accumulated displacement field, that is, by introducing diffeomorphic field accumulations.

As already mentioned above, the particularity of Morphons, compared to other nonparametric methods, is that the field computation (function Θ in (5)) is based on the local phase rather than intensity difference. In other words, knowing the phase difference between periodic signals of the same frequency allows the estimation of the spatial shift between them. Therefore, under the assumption that images can locally be considered as a sum of periodic signals, the computation of the local phase difference is equivalent to the estimation of the local displacement between images. This procedure is stabilized by the multiscale approach described in Section 3.2. Besides, Morphons combines the estimation of displacement vectors with a measure of the confidence we have in these estimations, resulting in a *certainty map*. Therefore, for Morphons, given two images *f* and *w* = *m*∘Δ, the displacement field estimation Θ is actually split into two quantities

(8) $$\begin{array}{c} D_{u}⟵\Theta_{D}\left(f,w\right),\\ c_{u}⟵\Theta_{c}\left(f,w\right), \end{array}$$

that is, respectively, an update of the displacement field along with an update of the certainty map. A similar split is also performed on subsequent operations Φ and Ψ.

Here are the details about the three steps {Θ, Φ, Ψ} of the pipeline of Section 3 for this specific registration, including our contribution to the field accumulation step.

### 4.1. Displacement Field Calculation

In Morphons, a displacement field is estimated thanks to the dephasing between the local phases of the fixed and the moving images. This local phase can be probed at a certain frequency and in a particular direction using quadrature filters [40]. More precisely, Morphons method uses a quadrature filter *h*_*η*_ of direction *η* ∈ ℝ^3^ (also called *loglets* [40]) defined in frequency by the polar separable function

(9) $$\begin{array}{c} H_{\eta }\left(\omega \right)=\chi_{{}_{+}}\left(\eta^{T}\omega \right){\left(\eta^{T}\hat{\omega }\right)}^{2}R\left(||\omega ||\right), \end{array}$$

where *ω* ∈ ℝ^3^ is the frequency vector, *χ*__+__(*λ*) = 1 if *λ* > 0 and 0 else, ||*ω*||^2^ = *ω*^*T*^*ω*, $\hat{\omega }=\omega /∥\omega ∥$ is the unit vector supporting *ω*, and *R* is a radial function centered on *ρ* > 0 and defined as *R*(*r*) = exp   [−ln ^2^(*r*/*ρ*)/ln 2] for *r* > 0.

Since their support corresponds to the half volume {*ω* ∈ ℝ^3^ : *η*^*T*^*ω* > 0} and since ${\left(\eta^{T}\hat{\omega }\right)}^{2}={\cos\;}^{2}\phi$ (with *ϕ* the angle separating *ω* and *η*), loglets can be seen as the analytic counterparts of the steerable filters introduced by Freeman and Adelson [41]. As a matter of fact, only a limited number of orientations *η* are necessary to cover the whole frequency plane. Typically, in 2D, these directions are taken as *η*_*k*_ = (cos *ϕ*_*k*_, sin *ϕ*_*k*_) with *ϕ*_*k*_ = *kπ*/4 for 0 ≤ *k* ≤ 3, and in 3D, *η* is taken as the 6 normal vectors {*η*_*k*_ : 0 ≤ *k* ≤ 5} to the faces of a hemi-icosahedron [42, 43]. Notice also that each filter *h*_*k*_(*x*) in the spatial domain is centered around the origin with a typical width given by 1/*ρ*.

Morphons take advantage of the following behavior. Given an image *f*, defining the filtering

(10) $$\begin{array}{c} q_{f}\left(x;k\right)\;=\;\left(f*h_{k}\right)\left(x\right), \end{array}$$

with ∗ the common convolution operation and the shorthand *h*_*k*_ = *h*_*η*_*k*__, we can write *q*_*f*_(*x*; *k*) = *A*_*f*_(*x*; *k*)*e*^*iϕ*_*f*_(*x*;*k*)^ since *q*_*f*_ ∈ *ℂ*. Therefore, by processing the warped image *w* similarly, the local phase difference can be computed as

(11) $$\begin{array}{c} \Delta \phi_{k}\left(x\right)=\arg\left(q_{f}\left(x;k\right)q_{w}^{*}\left(x;k\right)\right), \end{array}$$

with (·)* the complex conjugation and Δ*ϕ*_*k*_(*x*) = *ϕ*_*f*_(*x*; *k*) − *ϕ*_*w*_(*x*; *k*) the local *dephasing* between *f* and *w* in direction *η*_*k*_.

An important observation is that the nonnegative value

(12) $$\begin{array}{c} A_{f}\left(x;k\right)=\left|\left(f*h_{k}\right)\left(x\right)\right|=\left|\int_{{\mathbb{R}}^{3}}f\left(x^{\prime }\right)T_{x}{\bar{h}}_{k}\left(x^{\prime }\right)\text{d}x^{\prime }\right| \end{array}$$

represents also the correlation between *f*(*x*′) and the translated filter $T_{x}{\bar{h}}_{k}\left(x^{\prime }\right)={\bar{h}}_{k}\left(x^{\prime }-x\right)$; that is, the filter ${\bar{h}}_{k}\left(x^{\prime }\right)=h_{k}\left(-x^{\prime }\right)$ translated on *x*. If the image *f* was perfectly represented by the latter, that is, if we had locally *f*(*x*′) = *ch*_*k*_(*x*′ − *x*) for any *x*′ ∈ ℝ^3^ and some constant *c* ∈ ℝ, a displacement of *f* by a displacement field *D*(*x*) approximately constant over the support of $T_{x}{\bar{h}}_{k}$ would induce a dephasing Δ*ϕ*_*k*_(*x*) = *ρη*_*k*_^*T*^*D*(*x*) since the frequency vector of $T_{x}{\bar{h}}_{k}$ is −*ρη*_*k*_. An important implicit assumption is nevertheless that |*ρη*_*k*_^*T*^*D*(*x*)| < *π* since the dephasing is known up to modulo 2*π*. Moreover, only *η*_*k*_^*T*^*D* and not *D* can be determined, as another manifestation of the blank wall problem [44].

In practice, for most of *x*, *f*(*x*) is not perfectly represented by one filter but by a linear combination of them where the amplitude *A*_*f*_(*x*; *k*) measures the adequacy of the fit between *f*(*x*) and $T_{x}{\bar{h}}_{k}$. Consequently, the local update displacement *D*_*u*_(*x*) linking *f*(*x*) and *w*(*x*) = *f*(*x* + *D*_*u*_(*x*)) in each *x* ∈ ℝ^3^ is estimated by solving the weighted least square optimization

(13) $$\begin{array}{c} \Theta_{D}\left(f,w\right)=\underset{d\in {\mathbb{R}}^{3}}{\arg\;\min\;}\underset{k}{\sum }{\left[c_{k}\left(\rho \eta_{k}^{T}d-\Delta \phi_{k}\right)\right]}^{2}, \end{array}$$

where the *c*_*k*_(*x*) = *A*_*f*_(*x*; *k*)*A*_*m*_(*x*; *k*) are the *certainty map* of the filter *h*_*k*_. As explained above, *c*_*k*_ reflects for each voxel how reliable the field estimation is; that is, how contrasted the bandpass-filtered images are.

Numerically, the optimization in (13) is a standard weighted least square minimization; that is, it corresponds the minimization of the energy *E*(*d*) = ||*C*(*Nd* − Γ)||_2_^2^, using the diagonal matrix *C* = diag (*c*_1_,…, *c*_6_), the matrix *N* = (*η*_1_,…, *η*_6_)^*T*^, and the vector Γ = (Δ*ϕ*_1_,…, Δ*ϕ*_6_)^*T*^. An easy computation shows that the solution of (13) is then given by the Moore-Penrose pseudoinverse (*CN*)^†^ = (*N*^*T*^*C*^2^*N*)^−1^*N*^*T*^*C*, that is,

(14) $$\begin{array}{c} \Theta_{D}\left(f,w\right)={\left(CN\right)}^{†}C\Gamma , \end{array}$$

with Θ_*D*_(*f*, *w*) arbitrary set to 0 when (*N*^*T*^*C*^2^*N*) is not invertible.

Jointly to the estimation (13), a global certainty map associated to the quality of the estimation of Θ_*D*_ is defined as [43]

(15) $$\begin{array}{c} \Theta_{c}\left(f,w\right)=\underset{k}{\sum }c_{k}\left(x\right), \end{array}$$

that is, the sum of all certainty measures for each quadrature filter. This update of the certainty map must then be combined with an accumulated certainty computed from previous iterations (see Section 4.2).

In the multiscale approach described in Section 3.2, using the same quadrature filters at decreasing scales *H*_*η*_*k*__ is equivalent to estimating the phase of the bandpass-filtered image around increasing cutoff frequencies, that is, with *ρ* ← 2*ρ* each time *j* ← *j* + 1. This sustains the coarse-to-fine displacement estimation, that is, the computation of Θ_*D*_ and Θ_*c*_ on different scale bands *f*_*j*_ and *m*_*j*_ of *f* and *m*.

Convolutions with quadrature filters can be implemented efficiently in the Fourier domain thanks to the FFT and the convolution theorem. However, since the spatial extent of those filters is small, it is also possible to use efficient spatial convolutions with truncated kernels, as done in this study. As the local phase is invariant to local intensity scaling, the Morphons procedure is suitable for registering images with various contrast enhancements. Besides, some studies indicate that the phase extraction allows a fast convergence and a subvoxel precision in displacement estimation (e.g., see [39]).

### 4.2. Field Accumulation

In the original Morphons method, the accumulated field is computed as a weighted sum of the update field and the previous accumulated field, as used in damped optimization schemes. The weights are given by the certainty on the update field (*c*_*u*_, as computed from Θ_*c*_) and the accumulated certainty map (*c*_*a*_). As the certainty map must also be accumulated in order to reflect the confidence in all previous displacement computations, the accumulation step Φ must be divided into two operations Φ_*D*_ (field accumulation) and Φ_*c*_ (certainty accumulation):

(16) $$\begin{array}{c} \Phi_{D}\left(D_{a},D_{u},c_{a},c_{u}\right)=D_{a}+\frac{c_{u}}{c_{a}+c_{u}}D_{u}, \end{array}$$

(17) $$\begin{array}{c} \Phi_{c}\left(c_{a},c_{u}\right)=\frac{c_{a}^{2}+c_{u}^{2}}{c_{a}+c_{u}}, \end{array}$$

where in the last formula, similar to the field accumulation, the certainty map is updated by its own certainty [43].

However, as it was explained before, the addition of displacement fields is not really appropriate for accumulating spatial transformations, in contrast to composition. The compositive accumulation may also be damped using the certainty as a weighting factor

(18) $$\begin{array}{c} \Phi_{D}\left(D_{a},D_{u},c_{a},c_{u}\right)=D_{a}⊕\frac{c_{u}}{c_{a}+c_{u}}D_{u}. \end{array}$$

The (SSD-based) Demons registration is a nonparametric algorithm which performs the optimization of the SSD between images. In [27], a diffeomorphic field accumulation is proposed as improvement of the Demons method. The idea is to use an adaptation of the optimization method to Lie groups [45] in order to limit the possible solutions to diffeomorphic transformations. In practice, this is done by replacing the accumulation step of the Demons by an accumulation using the diffeomorphic flow exp () introduced in Section 2. This accumulation reads then

(19) $$\begin{array}{c} \Phi_{D}\left(D_{a},D_{u}\right)=D_{a}⊕\left(\exp\;\left(D_{u}\right)-\text{Id}\right), \end{array}$$

where the field exponential exp (*D*_*u*_) can be efficiently estimated using a small number of recursive compositions of the field *D*_*u*_ by itself. Consequently, the displacement field Φ_*D*_(*D*_*a*_, *D*_*u*_) is linked to the deformation operation Δ_*a*_∘exp (*D*_*u*_).

In the case of the Morphons, the accumulation step can be achieved in the same way. This will produce smoother fields than the traditional addition or composition. However, the accumulation step in the Morphons method involves a damping based on the certainty. Therefore, we propose the following accumulation step for diffeomorphic Morphons:

(20) $$\begin{array}{c} \Phi_{D}\left(D_{a},D_{u},c_{a},c_{u}\right)=D_{a}⊕\left(\exp\;\left(\frac{c_{u}}{c_{a}+c_{u}}D_{u}\right)-\text{Id}\right). \end{array}$$

Since exp (0  *D*) = Id for any vector field *D*, the accumulation fades away when *c*_*a*_ ≫ *c*_*u*_. The accumulation of the certainty map remains as explained previously in (17).

Notice that, because the field is discretized on a grid of voxels, interpolation is needed for computing the composition of two diffeomorphisms. Therefore, errors due to successive interpolations could potentially lead to noninvertible transformations. However, such problems were not observed in practical experiments using reasonable smoothing of the field.

### 4.3. Field Regularization

During the displacement estimation step, the relevance of local phase computation is estimated and used as weight for the accumulation. This certainty map may also be used for a smart regularization of the displacement field. Regularization is performed using a *normalized convolution* [46] of the field by a Gaussian kernel, taking into account the certainty map in order to put greater importance to high certainty locations. The certainty is also regularized in the same way as displacement field components in order to preserve the correspondence between the displacement vectors and their corresponding certainty.

Mathematically, given a positive function *h* and a filter *g* (typically a Gaussian kernel of variance *σ*_Ψ_ > 0), the normalized convolution of a (scalar) function *s* by *g* as involved by the normalization *h* is

(21) $$\begin{array}{c} s{*}_{h}g≜\frac{\left(hs\right)*g}{h*g}. \end{array}$$

This operation does not increase the maximum amplitude of the filtered function. Indeed, for a nonnegative kernel *g*, we show easily that ||*s*∗_*h*_*g*||_*∞*_ ≤ ||*s*||_*∞*_, with ||*s*||_*∞*_ = max_*x*_ |*s*(*x*)|. The accumulated displacement field *D*_*a*_ and subsequently the certainty map are therefore regularized thanks to this operation using for normalization the certainty map *c*_*a*_, that is,

(22) $$\begin{array}{cc} \Psi_{D}\left(D_{a},c_{a}\right) & =D_{a}{*}_{c_{a}}g,\\ \Psi_{c}\left(c_{a},c_{a}\right) & =c_{a}{*}_{c_{a}}g. \end{array}$$

Notice that, for computing Ψ_*D*_, the normalized convolution is performed separately on all components of the vector field.

This operation tends to propagate the displacement field from high certainty areas to areas which show less significant filter responses. Besides, by setting to zero the certainty outside the volume boundaries, normalized convolution cancels the influence of the padding strategy. This step produces a smooth version of the accumulated field that may reduce the accuracy of image matching resulting from the displacement estimation step, as it limits the possible solutions to smooth displacement fields.

However, if the iterative algorithm is to converge, the solution will be regular and invertible (except for large numerical errors), thanks to accumulation and regularization constraints, but it will also be (at least locally) optimal in terms of local phase difference. Indeed, as the phase is monotonic and smooth, a mismatch between local structures will automatically lead to nonzero field update with a high certainty value, which will tend to improve the displacement estimate and fit the structures together.

The Jacobian of the displacement field may be used as a criterion for validating the physical behavior of the deformation. Indeed, the Jacobian gives for each voxel the change in volume this voxel encounters during deformation. Jacobian indicates expansion when it is greater than 1, and compression when it is smaller than 1. A negative Jacobian means that the voxel is “inverted” (getting a negative volume), which is incompatible with the mass-preservation principle.

In the following, the diffeomorphic version of Demons and Morphons are denoted respectively D-Demons and D-Morphons.

## 5. Experiments and Results

The methods were first compared for several simple 2D virtual situations in order to demonstrate the interest in chosing the accurate registration method with respect to the images to be registered.

For the clinical validation, Morphons and D-Morphons registrations were first validated on a 10-phase point-validated pixel-based breathing thorax model (POPI-model) from the *Léon Bérard Cancer Center, Lyon, France* [4], in order to compare the D-Morphons to Morphons, Demons, and D-Demons in the case of intensity conservation between images. Then, it was applied to lung images with different contrast enhancements, in order to illustrate the benefit of a phase-based approach compared to traditional SSD-based registration methods in the case where intensities are not conserved between the images to be registered.

All simulations were performed using Linux, on a single processor Intel Core 2 (2.4 GHz). Our MATLAB implementation used for the prototyping of the methods was also used for simulation. Notice that no efforts were made for achieving good performances in terms of computational cost and memory requirements in the implementations used in this study. The local phase estimation was performed using convolutions with 9 × 9 × 9 quadrature filters. Less than 1 GB of RAM was required for registering two volumes of 256 × 256 × 100 voxels using all registrations. The time required for registering such images, using the parameters presented hereafter, was around 6 minutes for Demons, 42 for Morphons, 7 for D-Demons and 43 for D-Morphons. However, preliminary results based on a C++ implementation of the Morphons, which uses operations in the Fourier domain instead of convolutions (as done in our matlab implementation) and using 4 threads on a quad-core CPU, allowed a division of the computation time by 50, leading to Morphons registrations taking about one minute for such a typical image size.

### 5.1. Illustrative Virtual Experiments

**Figure 4:**
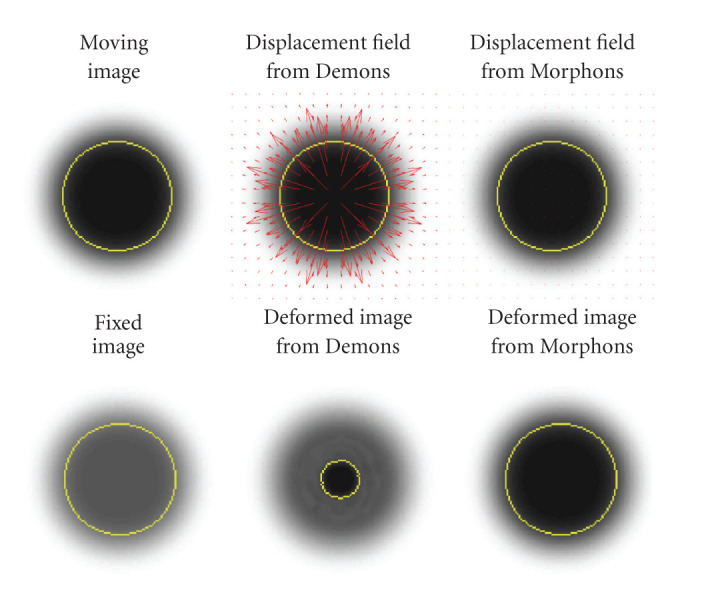
Results of the registration between 2 identical-sized blurred disks with different contrasts, using Demons and Morphons. In yellow: the contour of the disk. In red: the vector field resulting from the registration. The displacement field resulting from Morphons was very close to zero. Notice that the SSD is actually lower using Demons than Morphons. However, the SSD does not reflect the matching of the shapes, in opposition to the disk contour after warping.

Two 2D virtual experiments were performed. The first experiment, illustrated in Figure 4, is based on a virtual disk image after blurring. Two images of the same disk were created, the only difference being the scale of intensities (multiplication by 0.75). This experiment shows the interest in using a phase-based method (conventional Morphons in this example) while registering identical shapes with different contrasts, compared to an intensity-based method (conventional Demons).

**Figure 5:**
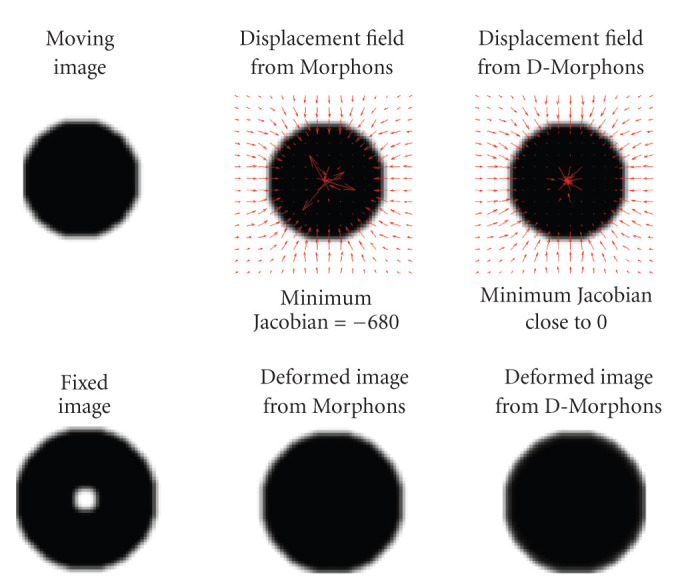
Results of the registration between 2 images using Morphons and D-Morphons registrations, illustrating the case where a structure (i.e., the bright hole at the center of the fixed image) is missing in the moving image. Both methods lead to deformed images very similar to the fixed image except for the central bright part (because it was not present in the moving image). The diffeomorphic method produced very low but still positive Jacobian values ((*J*) close to 0) in the center of the disk. Given that the field is defined on the pixel grid of the fixed image, this means that the surface of the central bright part (which disappears in the moving image) corresponds, as expected, almost to a singular point in the moving image. The conventional method, however, produced highly negative Jacobians in the central part, leading to the creation of areas that are “mirrors” of areas in the other image.

The second virtual experiment is based on two images of a disk (see Figure 5). In the fixed image, a disk of radius *r*_1_ + *r*_2_ was created, and a hole (disk of radius *r*_2_) was added in its center. In the moving image, a disk of radius *r*_1_ was created with the same intensity scaling as in the fixed image. This example illustrates the case where a structure is missing in one image compared to the other, as it may occur in practice (e.g., the problem of bowel gas in CT images of the abdomen). This experiment illustrates how the diffeomorphic version of the Morphons algorithm can prevent from producing negative volumes after registration, without increasing the smoothing by using a larger Gaussian regularization kernel.

### 5.2. Accuracy Assessment on a Breathing Thorax Model

The POPI model [4] is composed of 10 volumes reconstructed from a 4D respiration-correlated CT scan (RCCT) of the thorax, each volume corresponding to a particular phase of an average breathing cycle. 41 landmarks were identified by medical experts in each of the 10 images for registration validation.

Conventional Morphons, D-Demons, and D-Morphons were applied between a reference phase and the 9 others. For all methods, the number of scales was set to *J* = 8, with final resolution of 2 mm × 2 mm × 2 mm. The variance of the Gaussian kernel used for regularization was empirically set to twice the voxel size (*σ*_Ψ_^2^ = 2 voxels). For this experiment, a minimum of 10 and a maximum of 20 iterations was used at each scale. In between, the iterative process was stopped if the changes, measured in terms of SSD, were inferior to 0.01%. Such a convergence criterion was usually reached before the 20th iteration, supporting the fact that both Demons and Morphons behave like optimization methods.

The results were then compared with each other and with the results from a conventional Demons algorithm as used in [4]. The comparisons were achieved in terms of error in landmark position, SSD between images, harmonic energy, and minimum Jacobian.

- The landmark position error evaluates the ability of the registration in finding the physical motion of organs.
- The SSD between fixed and deformed images is a measure of the image matching according to the assumption of intensity conservation. It is computed as ∑_*x*_(*f* − *m*∘Δ)^2^.
- The harmonic energy [29, 32] of the displacement field *D* indicates how regular the field is and is computed as (1/2)∑_*x*_(||∇*D*_1_||^2^ + ||∇*D*_2_||^2^ + ||∇*D*_3_||^2^).
- The Jacobian of the field indicates the volume change of each voxel. Recall that negative values of the Jacobian correspond to inverted volumes, which is not acceptable in a physical point of view. The Jacobian is computed as det (*𝒥*), with *𝒥*_*ij*_ = ∂Δ_*i*_/∂*x*_*j*_ = *δ*_*ij*_ + ∂*d*_*i*_/∂*x*_*j*_, where *δ*_*ij*_ is Kronecker's delta (*δ*_*ij*_ = 1 if *i* = *j*, 0 else) and *d*_*i*_ is the *i*th component of the displacement field. In practice, the partial derivatives ∂*d*_*i*_/∂*x*_*j*_ can be computed using centered finite difference approximations.

The comparisons of landmark position errors (expressed in mm) resulting from the different registrations can be seen in Table 2 with, from left to right, the error in landmark position (norm of the difference) before registration, using Demons (values from the POPI website), Morphons, D-Demons, and D-Morphons. Position errors are noted as follows: mean/std (max). On average, for Morphons, D-Demons, and D-Morphons, the error in landmark position was equal or inferior to 1 mm, which is half the size of the voxels at the finest scale of the registration process.

**Figure 6:**
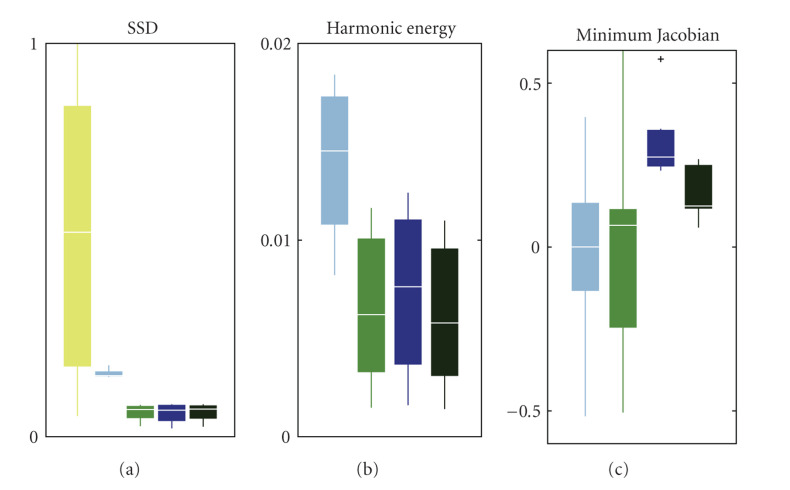
Results for the 9 registered phases of the POPI model. (a) Boxplots of the SSD before registration (in yellow) and after all 4 registrations. (b) Boxplots of the energy of deformation after all 4 registrations. (c) Boxplots of the minimum Jacobian after all 4 registrations. From (a) to (c), these registrations are Demons (light blue), Morphons (light green), diffeomorphic Demons (dark blue), and diffeomorphic Morphons (dark green). For each box, the center horizontal line represents the median value, the box goes from the lower quartile to the upper quartile, and the vertical lines represent the most extreme values within 1.5 interquartile range. The crosses represent outlier values.

Results showed that all registrations greatly improved the matching of intensities. The SSD between fixed and deformed image was similar for Morphons, D-Demons, and D-Morphons (see Figure 6). The harmonic energy of the fields resulting from these registrations was also comparable (see Figure 6).

The matching and the harmonic energy obtained by Demons (as presented by the authors of [4] on the POPI website) was slightly less good than for the 3 other methods. However, this is most likely due to the parameters used for registration (e.g., the number of scales, the variance for smoothing, etc.). In particular, for very similar images (first 2 phases of the RCCT), the algorithm was not able to find a smooth displacement field that reduced the SSD.

The minimum Jacobian of the displacement fields resulting from conventional methods gets down to −0.5 for both Demons and Morphons (see Figure 6), as, respectively, 67 and 460 voxels were inverted for the corresponding phase when applying the field on the moving image (which is composed of almost 6 mega voxels). However, when using diffeomorphic accumulation, the minimum Jacobian was raised to 0.2 for the Demons and 0.1 for the Morphons, showing that the diffeomorphic accumulation step prevented the field from inverting voxels.

### 5.3. Application to Images of the Thorax with and without Lodine Contrast Agent

The breathing-correlated motion of tumor is a typical feature of lung cancer that has to be dealt with in radiotherapy planning. RCCT images provide information about the tumor motion throughout the breathing cycle. From the different respiratory phases, an adequate margin around the tumor (the ITV, i.e., the Internal Target Volume) can be estimated, integrating thus all tumor positions through the respiratory cycle [20].

**Figure 7:**
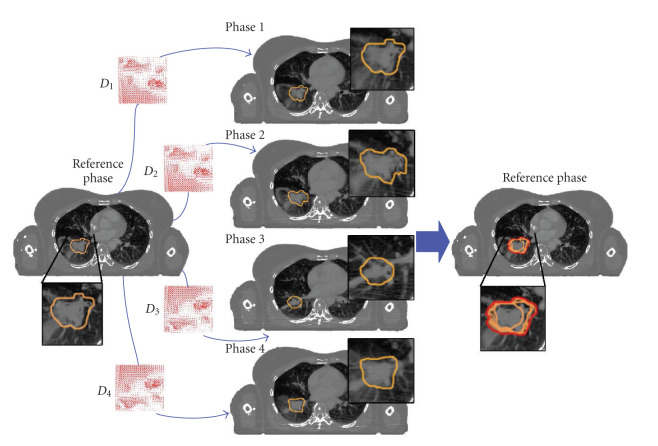
Schematic representation of the ITV creation (with only 4 phases). The CTV delineated on a reference image with contrast enhancement (on the left) is deformed towards every phases (middle) using displacement fields estimated by registration, and their union is taken as ITV (on the right).

However, the lack of contrast enhancement, as well as the high noise level and the presence of artifacts that characterize 4D RCCT, may significantly impair the accurate delineation of the target volumes on these images. More particularly, the iodine contrast agent is of prime importance to help at differentiating tumor extents from vascular structures in the centrally located lung tumors. In this context, the acquisition of a conventional contrast-enhanced CT (CE-CT) acquired during free breathing should be considered for the delineation task, while the 4D RCCT is used to estimate the motion range of the tumor during breathing. To automatize this process, the delineated tumor volume at the CE-CT can be deformed on the various respiratory phase images from the 4D RCCT using nonrigid registration to finally get the ITV, as illustrated on Figure 7.

The purpose of this experiment is to compare Demons and Morphons algorithms (conventional and diffeomorphic versions) for the registration between images with and without contrast enhancement, while keeping the same setting as for the POPI experiment.

A CE-CT scan of 3 lung cancer patients was acquired as well as a 4D RCCT scan at another time point. The first CT scan was taken in free breathing using an iodine contrast agent. The 4D RCCT scan was acquired without any contrast agent and was reconstructed into 10 phases. Histogram equalization was not able to correct for localized contrast differences between the CE-CT and RCCT phase images. For all 3 patients, Demons, Morphons, D-Demons, and D-Morphons were applied between each of the 10 RCCT images and the CE-CT, with the same registration parameters as for the POPI simulation.

**Figure 8:**
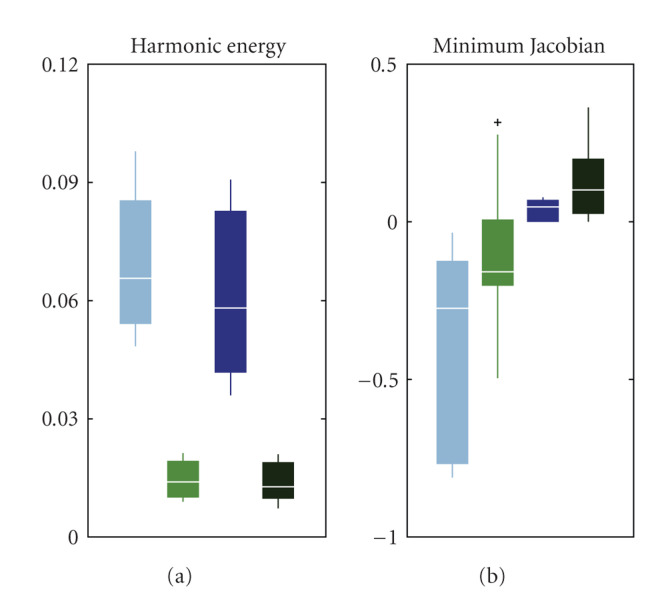
Results for the variable contrast experiment on 30 phases (3 patients with 10 phases each). (a) Boxplots of the energy of deformation after all 4 registrations. (b) Boxplots of the minimum Jacobian after all 4 registrations. From (a) to (b), these registrations are Demons (light blue), Morphons (light green), diffeomorphic Demons (dark blue), and diffeomorphic Morphons (dark green). For each box, the center horizontal line represents the median value, the box goes from the lower quartile to the upper quartile, and the vertical lines represent the most extreme values within 1.5 inter quartile range. The crosses represent outlier values.

The displacement fields resulting from these registrations were compared in terms of harmonic energy and minimum Jacobian (see Figure 8). The resulting images were compared in terms of SSD and mutual information.

**Figure 9:**
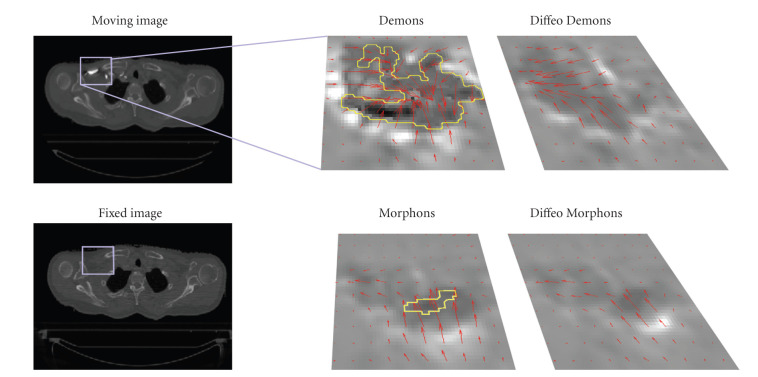
Illustration of negative Jacobians resulting from nondiffeomorphic registrations. Left: moving and fixed images. Right: fields resulting from registrations (red arrows) and their Jacobian (grayscale images). The negative Jacobians regions are contoured in yellow.

The harmonic energy of displacement fields resulting from Demons and D-Demons was quite higher than that with the Morphons and D-Morphons, and the minimum Jacobian of the displacement fields was positive only for registrations using the diffeomorphic accumulation. In the worst case, 7455 and 1114 voxels were inverted using respectively Demons and Morphons without diffeomorphic accumulation (on an image of 5 mega voxels). An example of area leading to bad transformations (with negative Jacobians) using conventional methods is depicted in Figure 9. D-Morphons lead to the smoothest transformation, with minimum Jacobian values around 0.2. These quite low values, however, were very sporadic within the image volume.

**Figure 10:**
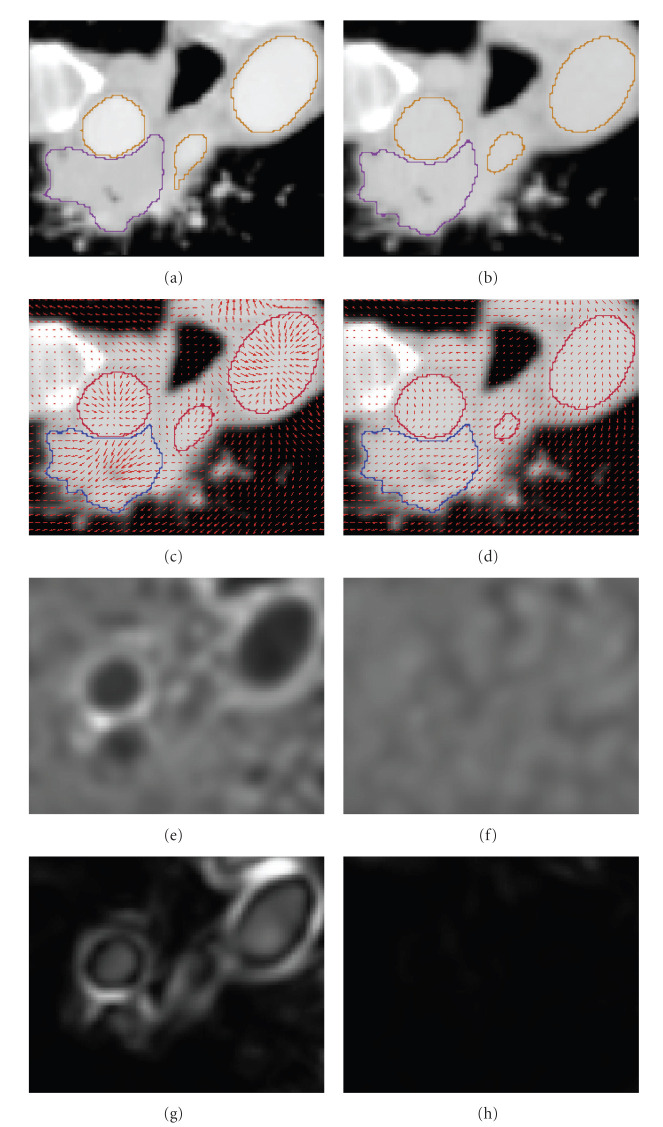
Illustration of the results for the variable contrast experiment. (a) and (b): Fixed image (left) and moving image (right). (c) and (d): Deformed image with deformed contours and displacement field resulting from D-Demons (left) and from D-Morphons (right). (e) and (f): Jacobian of the displacement field resulting from both registrations, represented using the same color scale. (g) and (h): Harmonic energy of the displacement field resulting from both registrations, represented using the same color scale.

We noticed that, unlike the results obtained with the POPI simulation, the SSD resulting from the Morphons and D-Morphons was a bit higher than the SSD resulting from Demons and D-Demons. However, as illustrated in the example of Figure 4, the SSD does not reflect the matching in variable contrast areas. On the other hand, no significant differences in terms of mutual information were observed between images resulting from the different registrations. This is likely due to the very low contrasts in the noncontrasted images within the regions corresponding to contrast-enhanced tissues in the other image whereas the main differences in terms of displacement field were located in these regions, as illustrated in Figure 10.

In order to illustrate the effect of the registration on contrast-enhanced tissues, one phase of the RCCT scan of one of the 3 patients was chosen as example. For this patient, the tumor was located close to contrasted tissues. The tumor and the blood vessels were delineated by a physician, on the contrast-enhanced scan and on one phase of the RCCT scan. The delineations on the phase image were deformed according to the fields resulting from the different registrations. The results are illustrated in Figure 10.

The change in volume due to warping was computed, as well as the harmonic energy inside the delineated stuctures and the difference between the center of mass of the tumor with and without registration.

The change in volume was very small when using a phase-based field computation for both the vessels (around 0%) and for the tumor delineations (around 1%), while it rose up to 23% for the vessels and to 6% for the tumor while using the Demons. In the same way, the harmonic energy and the error on the center of mass of the tumor were much smaller for the phase-based registration methods. These results are summarized in Table 3. One can notice that the diffeomorphic accumulation of the field in the Morphons did not change the results in terms of harmonic energy and volume changes compared to conventional Morphons. This is due to the fact that the displacement of the considered organs is small and smooth.

## 6. Discussion

The first medical experiment showed that D-Morphons and D-Demons lead to similar matching of both image intensities and anatomical landmarks. This shows that for monomodal registration of lung CT scans, the phase difference has an efficiency comparable to the efficiency of the SSD metric. Furthermore, the D-Morphons produced displacement fields as smooth as those obtained with D-Demons. In opposition to conventional Demons and Morphons, both diffeomporphic methods produced invertible displacement fields which are physically meaningful.

The second medical experiment illustrates the limitations in registering images with various levels of contrast enhancement with the Demons method. Indeed, the intensity matching resulting from Demons was better than that from Morphons, but the field was obviously wrong, as the Demons results in a global shrinking of the contrasted tissues (arteries) that does not reflect a proper anatomical behavior, but that is due to the fact that the Demons registration is based on the minimization of the SSD, which produces an improper displacement estimation when the intensities of identical tissues are different in the fixed and moving images. This mismatch between registered anatomical structures is clearly visible on Figure 10. As illustrated in the example of Figure 4, the field produced by Demons tries to match structures of same intensity, which do not correspond to identical anatomical structures because of the difference in contrast agent concentration. Therefore, the field resulting from Demons (see the field on the left part of Figure 10) is far less smooth than it should be and can lead to wrong deformation estimations as it illustrated in the example (see Table 3). In this case, the difference in intensity between the images with and without contrast enhancement lead to important volume changes for vessels and tumor by using Demons or D-Demons, while almost no changes in volume were observed for these tissues when using a phase-based approach. Besides, the harmonic energy inside these tissues shows that the field is much more smooth using the phase-based registration. It is important to notice that these effects are mostly limited by the regularization of the displacement field during the Demons and D-Demons registrations, and that they will still be worse if less regularization is used (smaller variance of the Gaussian kernel used for smoothing the displacement field). This is not the case for the fields produced by the Morphons and diffeomorphic Morphons, which are much smoother and preserve the anatomical topology even with contrast variations between images (see Figure 10). Notice that the reduction of the smallest segmentation that can be observed in the Morphons results is mostly due to interslices motion, as confirmed by the Jacobian close to 1 in this area that shows that there is no important volume changes within this segmented region. Finally, one can see that the invertibility of the displacement field is observed with both diffeomorphic registrations.

These results can be summarized by classifying the different registration strategies according to the smoothness (harmonic energy) and the invertibility (minimum Jacobian) of the resulting displacement fields (see Table 4) for the variable contrast experiment.

One can notice that the D-Morphons algorithm combines both advantages: the field is invertible and smooth, which suggests that it is likely a better estimation of the real transformation which is known to be smooth in this area.

## 7. Conclusion

The D-Morphons is a multiresolution registration algorithm which computes a diffeomorphic displacement field based on the minimization of the local intensity phase. The method managed to estimate the deformations in a breathing thorax, with an accuracy comparable to the accuracy of the D-Demons, and leads to the same requisite property of invertibility of the field. Moreover, the D-Morphons managed to accurately estimate the deformations between images with variable contrast, while the conventional SSD-based methods led to misalignment of anatomical structures affected by the contrast variation.

## Figures and Tables

**Table 1:** Multiscale nonparametric procedure.

**Inputs and parameters:**
(i) Images *f* and *m* defined on 1D4A2.
(ii) Number of scales *J*.
(iii) A stopping criterion *𝒮*.
(iv) Gaussian kernel variance *σ*_Ψ_^2^ of Ψ.
**Output:** The displacement field *D*_*a*_.
**Algorithm:**
(4) *Initialization*:
Set scale to *j* = *J* and initialize *D*_*a*_ = 0 on *𝒢*_*J*+1_.
(5) *Transfer on grid 𝒢*_*j*_:
Compute *m*_*j*_ = Down*_j_*(*m*), *f*_*j*_ = Down*_j_*(*f*),
and assign *D*_*a*_← Up(*D*_*a*_).
(6) While *𝒮* is false, do:
(i) *Warping*: *w* = *m*_*j*_∘Δ_*a*_
(ii) *Field computation*: *D*_*u*_ ← Θ(*f*_*j*_, *w*)
(iii) *Accumulation*: *D*_*a*_ ← Φ(*D*_*a*_, *D*_*u*_)
(iv) *Regularization*: *D*_*a*_ ← Ψ(*D*_*a*_)
(7) If *j* = 0, stop and return *D*_*a*_,
else, set *j* ← *j* − 1 and return to step (5).

**Table 2:** Results for the POPI experiment: error in landmark position.

RCCT phases	Original	Demons [4]	Morphons	D-Demons	D-Morphons
Phase 1	0.5/0.5 (2.4)	1.3/0.3 (1.8)	0.7/0.3 (1.6)	0.7/0.3 (1.6)	0.7/0.3 (1.6)
Phase 2	0.5/0.6 (2.6)	1.4/0.2 (2.1)	0.7/0.4 (2.1)	0.7/0.4 (1.6)	0.7/0.4 (2.1)
Phase 3	2.2/1.8 (6.6)	1.4/0.4 (2.3)	1.2/0.6 (2.5)	1.2/0.6 (2.5)	1.2/0.6 (2.4)
Phase 4	4.3/2.5 (10)	1.2/0.4 (2.3)	1.0/0.4 (2.2)	1.0/0.5 (2.5)	1.0/0.4 (2.2)
Phase 5	5.8/2.6 (12)	1.3/0.5 (2.6)	1.1/0.5 (2.7)	1.1/0.5 (2.5)	1.1/0.5 (2.8)
Phase 6	6.1/2.9 (14)	1.1/0.4 (2.0)	1.0/0.5 (2.1)	1.1/0.6 (2.8)	1.0/0.5 (2.1)
Phase 7	5.0/2.3 (12)	1.3/0.5 (2.4)	1.1/0.6 (2.8)	1.2/0.6 (2.7)	1.1/0.6 (2.8)
Phase 8	3.7/1.6 (6.2)	1.1/0.3 (1.7)	0.8/0.4 (1.9)	0.8/0.4 (1.8)	0.8/0.4 (1.8)
Phase 9	2.1/1.1 (4.5)	1.1/0.3 (1.9)	0.8/0.4 (2.0)	0.8/0.4 (1.7)	0.8/0.4 (2.0)

All phases	3.3/2.0 (14)	1.2/0.4 (2.6)	0.9/0.5 (2.8)	1.0/0.5 (2.8)	0.9/0.5 (2.8)

**Table 3:** Comparison of volume change, harmonic energy, and errors in center of mass of the delineations of the vessels and tumor on a single phase.

	Original	Demons	Morphons	D-Demons	D-Morphons
Volume change (vessels) [in %]		23	0	21	0
Volume change (tumor) [in %]		6	1	6	1
Harmonic energy (vessels) [×10^−3^]		89	8	69	8
Harmonic energy (tumor) [×10^−3^]		39	4	34	4
Error on COM (tumor) [in mm]	2.1	1.5	1.1	1.5	1.1

**Table 4:** Classification of the registration algorithms for variable contrast enhancement.

	Low harmonic energy	High harmonic energy
Invertible (*J*_min_ > 0)	D-Morphons	D-Demons
Noninvertible (*J*_min_ < 0)	Morphons	Demons
